# Supplementary material for: Prediction of The Mechanical Behavior of Polylactic Acid Parts with Shape Memory Effect Fabricated by FDM
Source: Polymers (Basel). 2023 Feb 25;15(5):1162. doi: 10.3390/polym15051162 (PMC10006872; doi:10.3390/polym15051162)
Supplement: Supplementary file 1 [file polymers-15-01162-s001.zip › polymers-2209713-supplementary.pdf]

Suplementary file (S1) for manuscript titled –

Prediction of the Mechanical Behavior of Polylactic Acid Parts with Shape Memory Effect Fabricated by FDM

Zhamila Issabaeva and Igor Shishkovsky*
Center for Materials Technologies, Skolkovo Institute of Science and Technology, 121205 Moscow, Russia
* Correspondence: i.shishkovsky@skoltech.ru; Tel.: +7-495-2801481

Table S1 All printing regimes.

№	D of the Nozzle, mm	Layer Height, mm	Infill Pattern	Infill Density, %	Temperature, °C
1	0.50	0.2	Grid	20	240
2	0.50	0.2	Grid	25	240
3	0.50	0.2	Grid	50	240
4	0.50	0.2	Grid	70	240
5	0.50	0.2	Grid	80	240
6	0.50	0.2	Line	20	240
7	0.50	0.2	Line	25	240
8	0.50	0.2	Line	50	240
9	0.50	0.2	Line	70	240
10	0.50	0.2	Line	80	240
11	0.50	0.2	Gyroid	20	240
12	0.50	0.2	Gyroid	25	240
13	0.50	0.2	Gyroid	50	240
14	0.50	0.2	Gyroid	70	240
15	0.50	0.2	Gyroid	80	240
16	0.50	0.3	Grid	20	240
17	0.50	0.3	Grid	25	240
18	0.50	0.3	Grid	50	240
19	0.50	0.3	Grid	70	240
20	0.50	0.3	Grid	80	240
21	0.50	0.3	Line	20	240
22	0.50	0.3	Line	25	240
23	0.50	0.3	Line	50	240
24	0.50	0.3	Line	70	240
25	0.50	0.3	Line	80	240
26	0.50	0.3	Gyroid	20	240
27	0.50	0.3	Gyroid	25	240
28	0.50	0.3	Gyroid	50	240
29	0.50	0.3	Gyroid	70	240
30	0.50	0.3	Gyroid	80	240
31	0.50	0.2	Grid	20	210
32	0.50	0.2	Grid	25	210

33	0.50	0.2	Grid	50	210
34	0.50	0.2	Grid	70	210
35	0.50	0.2	Grid	80	210
36	0.50	0.2	Line	20	210
37	0.50	0.2	Line	25	210
38	0.50	0.2	Line	50	210
39	0.50	0.2	Line	70	210
40	0.50	0.2	Line	80	210
41	0.50	0.2	Gyroid	20	210
42	0.50	0.2	Gyroid	25	210
43	0.50	0.2	Gyroid	50	210
44	0.50	0.2	Gyroid	70	210
45	0.50	0.2	Gyroid	80	210
46	0.50	0.3	Grid	20	210
47	0.50	0.3	Grid	25	210
48	0.50	0.3	Grid	50	210
49	0.50	0.3	Grid	70	210
50	0.50	0.3	Grid	80	210
51	0.50	0.3	Line	20	210
52	0.50	0.3	Line	25	210
53	0.50	0.3	Line	50	210
54	0.50	0.3	Line	70	210
55	0.50	0.3	Line	80	210
56	0.50	0.3	Gyroid	20	210
57	0.50	0.3	Gyroid	25	210
58	0.50	0.3	Gyroid	50	210
59	0.50	0.3	Gyroid	70	210
60	0.50	0.3	Gyroid	80	210
61	0.30	0.2	Grid	20	240
62	0.30	0.2	Grid	25	240
63	0.30	0.2	Grid	50	240
64	0.30	0.2	Grid	70	240
65	0.30	0.2	Grid	80	240
66	0.30	0.2	Line	20	240
67	0.30	0.2	Line	25	240
68	0.30	0.2	Line	50	240
69	0.30	0.2	Line	70	240
70	0.30	0.2	Line	80	240
71	0.30	0.2	Gyroid	20	240
72	0.30	0.2	Gyroid	25	240
73	0.30	0.2	Gyroid	50	240
74	0.30	0.2	Gyroid	70	240
75	0.30	0.2	Gyroid	80	240

76	0.30	0.3	Grid	20	240
77	0.30	0.3	Grid	25	240
78	0.30	0.3	Grid	50	240
79	0.30	0.3	Grid	70	240
80	0.30	0.3	Grid	80	240
81	0.30	0.3	Line	20	240
82	0.30	0.3	Line	25	240
83	0.30	0.3	Line	50	240
84	0.30	0.3	Line	70	240
85	0.30	0.3	Line	80	240
86	0.30	0.3	Gyroid	20	240
87	0.30	0.3	Gyroid	25	240
88	0.30	0.3	Gyroid	50	240
89	0.30	0.3	Gyroid	70	240
90	0.30	0.3	Gyroid	80	240
91	0.30	0.2	Grid	20	210
92	0.30	0.2	Grid	25	210
93	0.30	0.2	Grid	50	210
94	0.30	0.2	Grid	70	210
95	0.30	0.2	Grid	80	210
96	0.30	0.2	Line	20	210
97	0.30	0.2	Line	25	210
98	0.30	0.2	Line	50	210
99	0.30	0.2	Line	70	210
100	0.30	0.2	Line	80	210
101	0.30	0.2	Gyroid	20	210
102	0.30	0.2	Gyroid	25	210
103	0.30	0.2	Gyroid	50	210
104	0.30	0.2	Gyroid	70	210
105	0.30	0.2	Gyroid	80	210
106	0.30	0.3	Grid	20	210
107	0.30	0.3	Grid	25	210
108	0.30	0.3	Grid	50	210
109	0.30	0.3	Grid	70	210
110	0.30	0.3	Grid	80	210
111	0.30	0.3	Line	20	210
112	0.30	0.3	Line	25	210
113	0.30	0.3	Line	50	210
114	0.30	0.3	Line	70	210
115	0.30	0.3	Line	80	210
116	0.30	0.3	Gyroid	20	210
117	0.30	0.3	Gyroid	25	210
118	0.30	0.3	Gyroid	50	210

119	0.30	0.3	Gyroid	70	210
120	0.30	0.3	Gyroid	80	210

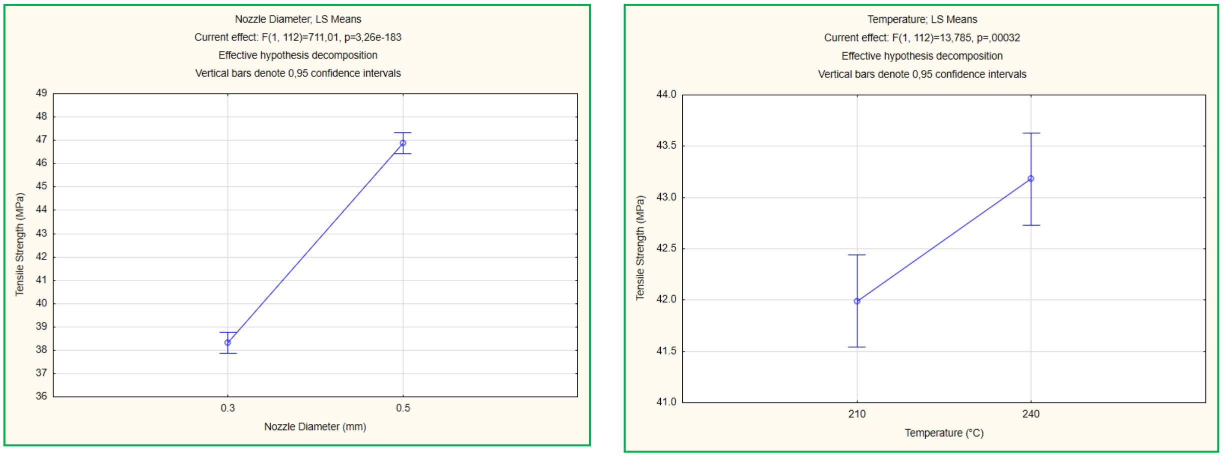

(**a**) (**b**)

Figure S1. The effects of a single parameter on strength: (**a**) the effect of nozzle diameter; (**b**) the effect of extrusion temperature.

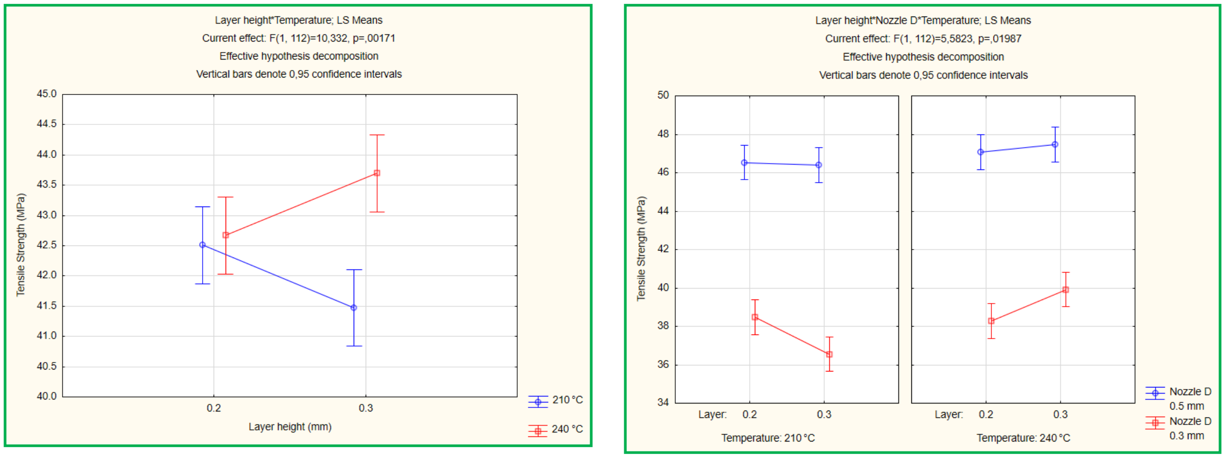

(**a**) (**b**)

Figure S2. The combined influence of two and three factors: (**a**) extrusion temperature and layer height; (**b**) extrusion temperature, nozzle diameter and layer height.
